# Two-dimensional strain-hardening membrane model for large deformation behavior of multiple red blood cells in high shear conditions

**DOI:** 10.1186/1742-4682-11-19

**Published:** 2014-05-13

**Authors:** Swe Soe Ye, Yan Cheng Ng, Justin Tan, Hwa Liang Leo, Sangho Kim

**Affiliations:** 1 Department of Biomedical Engineering, National University of Singapore, 9 Engineering Drive 1, Block EA #03-12, Singapore 117576, Singapore; 2 Department of Surgery, National University of Singapore, 1E Kent Ridge Road, Singapore 119228, Singapore

**Keywords:** Microhemodynamics, Blood rheology, Large deformation, Microfluidics

## Abstract

**Background:**

Computational modeling of Red Blood Cell (RBC) flow contributes to the fundamental understanding of microhemodynamics and microcirculation. In order to construct theoretical RBC models, experimental studies on single RBC mechanics have presented a material description for RBC membranes based on their membrane shear, bending and area moduli. These properties have been directly employed in 3D continuum models of RBCs but practical flow analysis with 3D models have been limited by their computationally expensive nature. As such, various researchers have employed 2D models to efficiently and qualitatively study microvessel flows. Currently, the representation of RBC dynamics using 2D models is a limited methodology that breaks down at high shear rates due to excessive and unrealistic stretching.

**Methods:**

We propose a localized scaling of the 2D elastic moduli such that it increases with RBC local membrane strain, thereby accounting for effects such as the Poisson effect and membrane local area incompressibility lost in the 2D simplification. Validation of our 2D Large Deformation (2D-LD) RBC model was achieved by comparing the predicted RBC deformation against the 3D model from literature for the case of a single RBC in simple shear flow under various shear rates (dimensionless shear rate G = 0.05, 0.1, 0.2, 0.5). The multi-cell flow of RBCs (38% Hematocrit) in a 20 μm width microchannel under varying shear rates (50, 150, 150 s^-1^) was then simulated with our proposed model and the popularly-employed 2D neo-Hookean model in order to evaluate the efficacy of our proposed 2D-LD model.

**Results:**

The validation set indicated similar RBC deformation for both the 2D-LD and the 3D models across the studied shear rates, highlighting the robustness of our model. The multi-cell simulation indicated that the 2D neo-Hookean model predicts noodle-like RBC shapes at high shear rates (G = 0.5) whereas our 2D-LD model maintains sensible RBC deformations.

**Conclusion:**

The ability of the 2D-LD model to limit RBC strain even at high shear rates enables this proposed model to be employed in practical simulations of high shear rate microfluidic flows such as blood separation channels.

## Introduction

The transport behavior of blood in microcirculatory flows can be characterized by the mechanical response of the two major constituents of the blood mixture to the fluidic stresses driving the bulk flow. The first major constituent of blood is plasma, which under physiological conditions has Newtonian properties and a viscosity similar to water. The second major component is the red blood cells (RBCs) that make up about 35% to 45% of the systemic blood volume for an average individual [1]. The RBC phase contributes significantly to the complex behavior of blood in micro-flows, such as shear-thinning, the Fahraeus effect and the Fahraeus-Lindqvist effect. Due to these significant contributions to blood microrheology, the mechanical characteristics of RBCs have been studied extensively. The shape of the RBC can be defined by a membrane that separates the inner fluid (cytoplasm) from the suspending fluid (blood plasma). The most notable properties of the RBC membrane are its hyperelastic and viscoelastic response to high shear stress, membrane area incompressibility and the ability to recover its initial shape with the removal of external stress [2,3].

In accordance with these properties, many previous studies [4-12] have been undertaken to describe the mechanical behavior of RBCs *in silico*. In these studies, the various numerical discretization techniques for the RBC model range from the mesoscale approach with cytoskeletal network models [4,5] and particle method models [6] to the macroscopic approach of viscoelastic spring network models [7,8], finite element method (FEM) models [9] and the boundary integral method (BIM) models [10]. With regards to the macroscopic RBC modeling approach, most previous studies have assumed either the neo-Hookean [10-13] or the Skalak constitutive relations [3,14-16] to describe the non-linear stress-strain shear response of the RBC membrane deformation. In general, the membrane shear response is considered to be the most dominant deformation modality in the RBC membrane mechanical response. Although 3D simulations based on the macroscopic RBC models have previously been performed, the usefulness of such 3D simulations may be very limited due to the extremely high computational cost; consideration of the RBC interactions (aggregation and disaggregation) in high hematocrit flows for a 3D simulation is not feasible without employing sophisticated parallel computing techniques. Consequently, many previous numerical studies have instead utilized 2D RBC models to simulate physiological blood flows [6-8,13,17].

In the 2D modeling approach, the neo-Hookean and Skalak constitutive relations have been reformulated for 2D by removing a principle strain direction from the original 3D formulation [18]. However, employing the 2D formulations without modifying the effective moduli can overpredict the extent of deformation in the RBC membrane due to the disappearance of the Poisson’s effect contributed by the second principle strain direction. Furthermore, one important membrane feature that has been considered in the 3D simulation but not in the 2D simulation is the surface area incompressibility of the RBC membrane attributed by its incompressible lipid bilayer. Essentially, the extensional resistance of the RBC cross-section is not entirely a result of the membrane’s shear resistance. Consequently, the significant loss of these two deformation modalities in the 2D simulation severely limits the accurate prediction of the RBC 2D cross-sectional profiles under complex flow conditions such as high shear rates, crowded cell-cell interactions under high hematocrits and multi-directional RBC strain. This inaccuracy has limited 2D studies in the literature to the low flow regime models where shear rates are typically less than 300 s^-1^[7,8].

In the present study, we propose a modification of the 2D neo-Hookean relation to compensate for the apparent softening of the RBC membrane in 2D. The modified membrane model is coupled with the lattice Boltzmann method (LBM) flow solver through the immersed boundary method (IBM) [19]. For the membrane model development, a large deformation scaling coefficient is applied to the neo-Hookean membrane model and its several complementary constitutive relations to introduce a strain hardening effect on the RBC membrane at high strain rates.

## Numerical model

### Lattice Boltzmann method (LBM)

LBM simplifies the original Boltzmann equation by discretizing time, space and momentum [20,21] through the employment of a lattice grid. Its mesoscopic nature arises from the fact that it considers microscale kinetic conditions of the fluid particles in relation to the macroscopic variables such as continuum mass and momentum. In a 2D discretization of space, the microfluxes are quantified on the square lattice in 9 directions under the D2Q9 approach [22]. The key concept of the LBM approach is that the microstates in the mesosystem evolve as a result of the macroscale (continuum) conditions and the evolution is conducted in key stages known as the streaming and collision stages. To represent the statistical contribution of the 9 directions, the objective quantifiers of the microstates are given by the density distribution function in the LBM formulation, summation of the 9 density distribution functions at a lattice grid point gives the local fluid density of the macroscopic continuum.

The LBM equation with a general body force term [23] is expressed as:

(1) $$f_{i}\left(\vec{x}+{\vec{c}}_{i}\mathrm{\Delta t},t+\mathrm{\Delta t}\right)-f_{i}\left(\vec{x},t\right)=-\frac{1}{\tau }\left[f_{i}\left(\vec{x},t\right)-f_{i}^{\mathrm{eq}}\left(\vec{x},t\right)+\mathrm{\Delta t}B_{i}\right]$$

where $f_{i}\left(\vec{x},t\right)$ is the density distribution function of the particles moving with lattice velocity ${\vec{c}}_{i}$ at position $\vec{x}$ and time *t*, Δt is the lattice time step, *τ* is the relaxation time and *B*_*i*_ is the body force term discretized in the 9 lattice directions denoted by the subscript *i*. In Eq. (1), left-hand side terms represent the streaming stage for the 9 density distribution functions *f* denoted by the subscript *i*. The first right-hand side term represents the collision contribution to the distribution functions [24] whereby microstates are disturbed from their equilibrium states which is given by the equilibrium density distribution function $f_{i}^{\mathrm{eq}}\left(\vec{x},t\right)$:

(2) $$f_{i}^{\mathrm{eq}}\left(\vec{x},t\right)=\omega_{i}\rho \left[1+\frac{\vec{u}\cdot {\vec{c}}_{i}}{c_{s}^{2}}+\frac{1}{2}{\left(\frac{\vec{u}\cdot {\vec{c}}_{i}}{c_{s}^{2}}\right)}^{2}-\frac{u^{2}}{2c_{s}^{2}}\right]$$

where $\vec{u}$ is the continuum fluid velocity, *ω*_*i*_ is the weight factor, taking the value of 4/9 for *ω*_0_, 1/9 for *ω*_1-4_, and 1/36 for *ω*_5-8_. The lattice speed of sound is given by the form $c_{s}=h/\left(\sqrt{3}\mathrm{\Delta t}\right)$ where *h* is the lattice cell (space step) size. The second right-hand side term, which is the aforementioned body force term, includes all the fluid-structure interaction (FSI) forces between the suspending fluid and the RBC membrane. Calculation of the body force term is given as:

(3) $$B_{i}=\left(1-\frac{1}{2\tau }\omega_{i}\right)\left[\frac{{\vec{c}}_{i}-\vec{u}}{c_{s}^{2}}+\frac{{\vec{c}}_{i}\cdot \vec{u}}{c_{s}^{4}}{\vec{c}}_{i}\right]\cdot {\vec{F}}_{f}$$

where ${\vec{F}}_{f}$ is the total FSI body force acting on the lattice (fluid) grid node due to on-membrane forces.

Lastly, the macroscopic constrains imposed on the lattice Boltzmann system through continuum fluid density *ρ* and continuum velocity $\vec{u}$ can be obtained from the fundamental equivalency between the macrostate quantities and the summation of microstate quantities (conservation of mass and momentum):

(4) $$\rho =\sum_{i}f_{i},\;\vec{u}=\sum_{i}\left(f_{i}{\vec{c}}_{i}+0.5{\vec{F}}_{f}\mathrm{\Delta t}\right)/\rho$$

The validity of the LBM transport equation is well-established since the Navier-Stokes equations can be derived from Eq. (1) through the Chapman-Enskog expansion [25]:

(5) $$\frac{\partial \rho }{\partial t}+\nabla \left(\rho \vec{u}\right)=0$$

(6) $$\frac{\partial \vec{u}}{\partial t}+\left(\vec{u}\cdot \nabla \right)\vec{u}=-\frac{1}{\rho }\nabla P+\nu \nabla^{2}\vec{u}$$

where $v=\left(\tau -\frac{1}{2}\right)c_{s}^{2}\mathrm{\Delta t}$ and $P=c_{s}^{2}\rho$.

### Immersed boundary method (IBM)

The IBM [26] was employed in our simulation to account for the acceleration effect of a moving boundary on the fluid through the application of a distributed force density evaluated from the boundary’s constitutive laws. In this method, the fluid domain is represented by an Eulerian mesh where the globally referenced coordinates of the fluid grid point are given by $\vec{x}$. The RBC membrane boundary is represented by a Lagrangian mesh with body-fixed coordinates $\vec{s}$ used for the membrane force calculations. The globally referenced coordinate location of a node on the moving membrane can be given by $\vec{X}\left(\vec{s},t\right)$.

To satisfy the non-slip boundary condition between the membrane and the adjacent fluid, the membrane inherits the same velocity ${\vec{u}}_{f}$ as the fluid. Since a Lagrangian membrane mesh node does not always coincide perfectly with the Eulerian fluid grid points, the membrane velocity ${\vec{u}}_{m}$ is interpolated from the neighborhood of fluid grid points around the membrane node:

(7) $${\vec{u}}_{m}=\sum_{f}{\vec{u}}_{f}\left(\vec{x},t\right)\phi_{f}\left({\vec{x}}_{f}-{\vec{X}}_{m}\right)$$

where $\phi_{f}\left({\vec{x}}_{f}-{\vec{X}}_{m}\right)$ is the interpolation function and subscripts *m* and *f* denote the membrane node and fluid grid point indices, respectively. It is given by the discrete delta function:

(8) $$\phi_{f}\left(\vec{r}\right)=\frac{1}{4h^{2}}\left(1+\cos\frac{\pi r_{x}}{2h}\right)\left(1+\cos\frac{\pi r_{y}}{2h}\right),|r_{x}|\leq 2h\;\mathrm{and}\;|r_{y}|\leq 2h$$

In the coupling stage of the IBM routine, the resulting node displacement in a deformation induces a reaction force ${\vec{X}}_{m}\left(\vec{s},t\right)$ back onto the Eulerian fluid through the spreading of a fluid force density ${\vec{F}}_{f}\left(\vec{x},t\right)$ given by:

(9) $${\vec{F}}_{f}\left(\vec{x},t\right)=\sum_{m}{\vec{F}}_{m}\left(\vec{s},t\right)\phi_{m}\left({\vec{x}}_{f}-{\vec{X}}_{m}\right)$$

where $\phi_{m}\left({\vec{x}}_{f}-{\vec{X}}_{m}\right)$ is the spread function, given by the same discrete delta function in Eq. (8).

The force density spreading and membrane node velocity interpolation are performed on a 4*h* × 4*h* region [26]. To describe the different properties of blood plasma and cytoplasm within the RBCs, an indicator field approach was employed to update the moving topology of the plasma domain and RBC interior cytoplasm domain at every time-step. By utilizing our recently developed method (flood-fill method) [27], we have assigned a viscosity of 6.0 and 1.2 cP to the cytoplasm and plasma respectively, thereby capturing the viscoelastic response of the membrane deformation due to the RBC interior-exterior viscosity ratio. The details of the flood-fill algorithm used to update the fluid properties during the simulations can be found in our earlier work [27].

### RBC model

**Figure 1:**
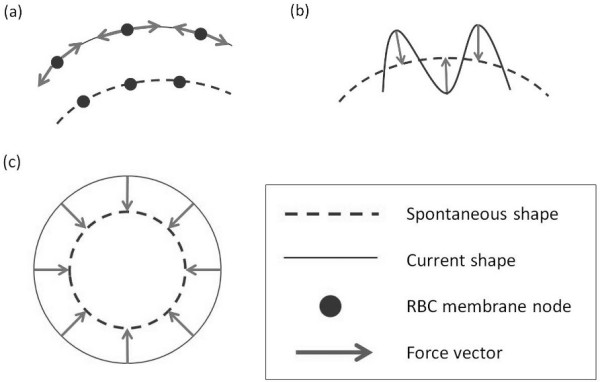
**Schematic diagram of the 3 types of membrane forces implemented in the RBC model. (a)** Extensional force which restricts the elongation of the RBC perimeter. **(b)** Bending force which controls the local curvature of the RBC. **(c)** Dilation force which maintains the RBC internal area.

The shape of the RBC is maintained by four main deformation modalities which govern the mechanics of the membrane. Figure 1 summarizes all the internal forces considered in the RBC membrane for our 2D model. The RBC circumference is discretized into a Lagrangian mesh with several membrane nodes connected by non-linear spring segments. The internal forces considered for the RBC model are the membrane shear, bending and RBC volume conservation forces.

#### Membrane in-plane shear

The constitutive shear behavior of the RBC membrane is non-linear and exhibits a degree of strain-hardening. Under the 2D neo-Hookean model formulation [27-29], the membrane shearing stress-strain relation is linear in the small deformation range and non-linear in the large deformation range as shown in Eq. (10):

(10) $$\tau =\frac{E_{S}}{\lambda^{3/2}}\left(\lambda^{3}-1\right)\;\mathrm{where}\;\lambda =\frac{l_{m}}{l_{0}}$$

where *E*_s_ is the shear elastic modulus of the membrane and *λ* is the stretch ratio on the local membrane segment given by the ratio of the current membrane segment length *l*_*m*_ over the initial membrane segment length *l*_0_. The shear elastic modulus in this study is set at 6 × 10^-3^ dyn cm^-1^, which is within the physiological range for RBC elastic properties [30].

As discussed earlier, the limitation of applying the 2D neo-Hookean model is the unrestricted stretching of membrane perimeter (circumference). Previous studies [31,32] have shown that use of the neo-Hookean model alone is unable to restrict the membrane surface area changes in 3D capsules from exceeding 7.8% at shear rates above 300 s^-1^. Conversely, the changes in global RBC membrane surface area should be less than 5% under physiological conditions due to the incompressibility of the lipid bilayer in the membrane [31]. The stretching of the RBC membrane is expected to be overpredicted for the case of 2D simulations where the extension of a 1D surface (line) is unrestricted due to the lack of the Poisson’s effect from the second principle strain direction.

In the present study, a 2D large deformation (LD) neo-Hookean model is proposed to account for membrane area incompressibility observed in experimental and 3D simulation studies and to also compensate for the lack of the dilatory restriction. The neo-Hookean model for 2D capsules is therefore modified by the large deformation scaling coefficient *α*, which is a function of the local RBC membrane stretch ratio *λ*. The 2D-LD neo-Hookean model developed in this study can be presented as follows:

(11) $$\tau =\frac{E_{S}}{\lambda^{3/2}}\left(\lambda^{3}-1\right)\alpha \left(\lambda \right)$$

where α(*λ*) is given by:

(12) $$\alpha \;\left(\lambda \right)=1+D_{\mathrm{LD}}\left(e^{{}^{\lambda \beta_{\mathrm{LD}}}}-e^{{}^{\beta_{\mathrm{LD}}}}\right)$$

where *D*_*LD*_ and *β*_*LD*_ are constants. It is of note that the value of *α* from Eq. (12) approaches unity at very low strains and Eq. (11) reverts back to its original form in Eq. (10) under such conditions.

#### Membrane bending

To control the curvature of the RBC, a bending force is implemented on the RBC membrane as follows:

(13) $$T_{b}\left(\kappa \right)=E_{b}\left(\kappa -\kappa_{0}\right)\alpha \left(\lambda \right)$$

where *E*_b_ is the bending modulus of the RBC membrane, *κ* is the current membrane curvature and *κ* is the spontaneous curvature of the un-deformed RBC. The bending force is similarly scaled by the large deformation coefficient *α* presented in Eq. (12). The scaling of the membrane flexural resistance is necessary to prevent membrane buckling under high compression which instigates numerical instabilities.

#### Cytoplasmic volume conservation

As the membrane shear and bending models only consider the surface of the RBC membrane, the RBC internal volume (internal area in 2D) is not implicitly conserved; since the bulk of cytoplasm in an RBC does not exit its membrane, the internal volume conservation needs to be enforced in the deformation dynamics of the membrane. Hence, an interior pressure force *p*_*int*_ is introduced to act on the RBC membrane, thereby strictly imposing the conservation of the RBC internal volume and mass. The pressure penalty model for a 2D capsule can be expressed as follows:

(14) $$p_{\mathrm{int}}=k_{p}\left(1-A/A_{\mathrm{ref}}\right)\;\alpha \left(\lambda_{C}\right)\;\mathrm{where}\;\lambda_{C}=\frac{L_{C}}{L_{0}}$$

where *k*_*p*_ is the incompressibility coefficient and the argument *λ*_*C*_ is the RBC perimeter extension ratio given by ratio of the current RBC circumference *L*_*C*_ over the initial circumference of the circular RBC *L*_0_. *A* is the internal area of the deformed RBC, and *A*_ref_ is the initial internal area of the RBC. The internal area of the RBC is calculated using Green’s theorem:

(15) $$A=\int_{C}x_{m}dy_{m}-y_{m}dx_{m}$$

where *x*_*m*_ and *y*_*m*_ are the coordinates of the points on the RBC membrane curve C.

By taking a sufficiently large incompressibility coefficient *k*_*p*_ and by considering the growing restriction under large deformation using the α(*λ*_*C*_) term, we can engage a sufficiently large internal pressure *p*_*int*_ to maintain the constant RBC size in the simulation. The maintenance of a constant RBC area is a necessary constraint in order to satisfy the conservation of cytoplasmic mass in the channel flow. Accordingly, the cytoplasmic mass is not allowed to arbitrarily swell or disappear from the movement of the RBC membrane. Consequently, by including the cytoplasmic conservation in our model, the RBC area and 2D hematocrit in the channel can be maintained at a constant value throughout the entire simulation.

#### RBC-RBC interaction

Blood microrheology can be significantly affected by the cell-to-cell interactions occurring within the carrying vessel. RBCs in physiological flows can aggregate due to the presence of large molecules such as fibrinogen, this attraction between aggregating cells typically occurs over the sub-micron to nano length-scales. Conversely, RBCs can repel one another when brought within interacting distance of the glycolayx due to steric hindrance and repulsion between like negative charges on the RBC membranes. In the present study, the depletion theory is employed to describe the aggregation and repulsion between the RBC membranes [33]. The total interaction energy *φ* can be expressed using the Morse-type potential energy function [34]:

(16) $$\phi \left(r\right)=D_{e}\left[e^{2\beta \left(r_{0}-r\right)}-2e^{\beta \left(r_{0}-r\right)}\right]$$

where *r* [μm] is the separation distance between the pairing membrane nodes and *r*_0_ [μm] is the zero force distance specified in the model. *D*_*e*_ [μJ μm^-2^] is the surface energy and *β* [μm^-1^] is the scaling factor that determines the rate of interaction energy decay with distance. In this study, *r*_0_, *β* and *D*_*e*_ were set with the values of 0.49, 3.84 and 1.3 × 10^-7^ respectively as reported in previous studies [27,35]. The total interaction force between the membrane nodes is expressed as the negative derivative of the interaction potential from Eq. (16):

(17) $$F_{\mathrm{agg}}\left(r\right)=-\frac{\partial \varphi }{\partial r}\alpha \left(r\right)=2\beta D_{e}\left[\;e^{2\beta \left(r_{0}-r\right)}-e^{\beta \left(r_{0}-r\right)}\right]\alpha \left(r\right),\;\mathrm{where}\;\alpha \left(r\right)=\left\{\begin{array}{c} 1,\;r>r_{0}\\ \alpha \left(\lambda_{C}\right),\;r\leq r_{0} \end{array}\right)$$

In Eq. (17), a negative *F*_*αgg*_ value when *r* > *r*_0_ indicates an aggregating (attraction) force while a positive value when *r* ≥ *r*_0_ represents a repulsion force. Additionally, the LD coefficient scales only the repulsion force between pairing RBC membranes to prevent cell to cell overlap from the increase in internal forces from the shear, bending and dilatory modalities.

**Figure 2:**
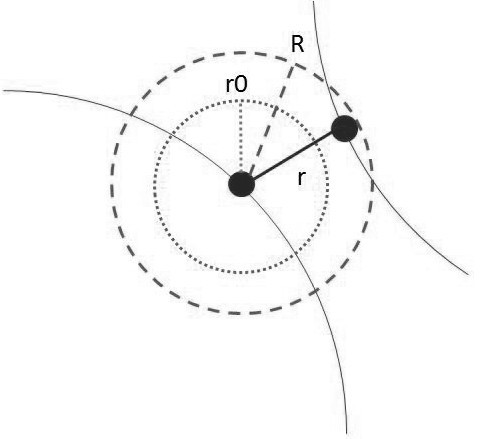
**Schematic diagram of an RBC membrane node with the two regions of interest.** Beyond the active distance R, the interaction force decays to zero.

The interaction between RBCs as dictated by Eqs. (16) and (17) is illustrated in Figure 2. A querying region is defined around every RBC membrane node to locate the nearest membrane node on the neighboring RBC for the paired interaction. When the distance between the paired membrane nodes is less than *r*_0_, the node-pair experiences a repulsion force. However, when the distance between the two nodes is within the *R* to *r*_0_ range, the node-pair experiences an attraction force.

### Simulation setup

**Figure 3:**
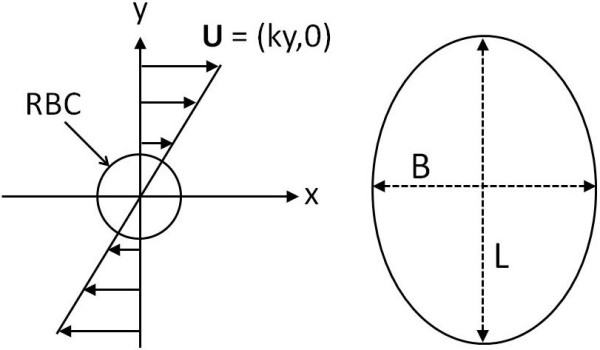
Schematic illustration of a simple shear flow condition on the suspended circular RBC (left) and an elliptical shape of the deformed RBC (right).

We have performed two sets of simulations: 1) a single cell in a simple shear flow to validate our large deformation (2D-LD) model and 2) multiple cells in a channel flow. In the single cell study, a velocity field of *u* = *ky* is imposed where the strain rate *k* can be obtained by the simple relation *k = U/Y. Y* is the half-height of the simulation domain and *U* is the maximum magnitude of the velocity at the top and bottom of the simulation domain as presented in Figure 3. The deformation of the capsule is described by the Taylor deformation index *D*_xy_ which is given by:

(18) $$D_{\mathrm{xy}}=\frac{L-B}{L+B}$$

where *L* is the major diameter of the RBC and *B* is the minor diameter as shown in Figure 3. Notably, this characterization of the RBC deformation only works for RBCs that adopt a 2D ellipse profile and the value of *D*_*xy*_ is highly sensitive to the major and minor diameters at low deformation states.

In our multi-cell channel flow simulations, we highlight the efficacy of the 2D-LD model for predicting RBC deformation under moderate to high shear rates by comparing its deformation result against the 2D neo-Hookean model. Furthermore, we investigated the importance of the scaling relationship for the strain-hardening between the four deformation modalities acting on the RBC membrane. To achieve these comparative investigations, we utilized three sets of conditions that have been summarized in Table 1. Case I represents the original neo-Hookean model in 2D since the LD scaling coefficient was not implemented to any constitutive model. In Case II, the LD scaling coefficient was only applied to the membrane shear constitution. This approach is similar in concept to earlier studies where the non-linear stiffening behavior is not considered for the membrane bending and cell-to-cell interactions [18]. Finally, Case III represents the full 2D-LD model whereby the LD scaling coefficient was applied to all four RBC mechanical constitutions.

**Table 1:** Multi-cell channel flow simulations

**Case**	**LD scaling applied**			
	**Shear**	**Bending**	**Vol.**	**Cell-cell**
			**conservation**	**interaction**
I	X	X	X	X
II	√	X	X	X
III	√	√	√	√

In the initial condition, twelve circular RBCs were suspended in a periodic arrangement inside a channel of 80 μm by 20 μm to achieve a physiological hematocrit level (38%). The circular RBC cross-sectional profile was chosen for simulation as this 2D profile represents the most extreme shearing orientation for the RBCs in a narrow channel. Pressure boundary conditions were prescribed for the pressure-driven flow to obtain the pseudoshear rates (mean velocity/channel width) of 50, 150, and 500 s^-1^ for each of the three cases. Periodic translations were implemented on the RBCs at the inlet and outlets such that RBCs leaving the simulation domain re-enter from the inlet, thereby maintaining the same number of 12 RBCs for the entirety of the simulation.

## Results and discussion

### 2D large deformation (2D-LD) model validation

To characterize the deformation of the 2D RBC in relation to the shear condition, the dimensionless shear rate *G* was used. *G* provides a normalized indication of the stress condition on the cell by comparing the estimated fluidic shear stress applied on the RBC membrane (numerator) to the inherent elastic property of the membrane (denominator) [12]:

(19) $$G=\frac{\mathrm{\mu ka}}{E_{s}}$$

where *μ* is the dynamic fluid viscosity, *k* is the shear rate, and a is the equivalent radius of the RBC. Breyiannis and Pozrikidis [11] have compared the deformation of 2D solitary capsules against the deformation of 3D spherical capsules and have reported a good correlation. They established a *D*_xy_ correspondence between the *G* values for circular capsules and 3D spherical capsules by using their cross-sectional profiles. Consequently, the empirical equation relating the 3D *G* to 2D *G* was reported to be:

(20) $$G_{2D}=-0.008417+0.45073G_{3D}+0.75662G_{3D}^{2}$$

**Figure 4:**
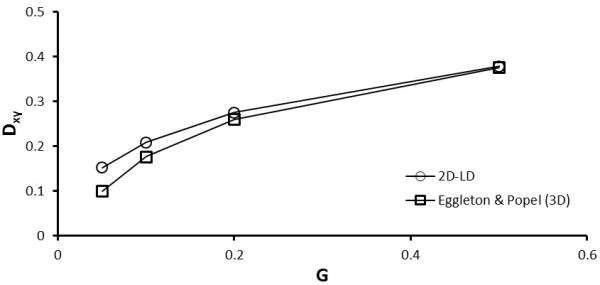
**Validation of the LD neo-Hookean (2D-LD) model on a circular capsule against the 3D spherical capsule results**[31].

Based on this relation, we have validated our 2D capsule deformation results with the 3D spherical capsule deformation reported in a previous study by Eggleton and Popel [31]. Figure 4 shows the results of the validation. Comparing the *D*_xy_ values obtained for the dimensionless shear rates *G* of 0.05, 0.1, 0.2 and 0.5, we observed a reasonable correspondence between our 2D results and the 3D model results of Eggelton and Popel. While the discrepancy is close to 50% at the lowest shear rate, the 2D-LD model can sufficiently limit the RBC deformation to agree with the 3D model data at higher shear rates. It is likely that the low shear rate discrepancy arises as a result of Eq. (20) presenting non-sensible G values for the 2D equivalent at very low shears. For example, conversion of the 3D G at a value of 0.01 using Eq. (20) results in a 2D G value of -0.00383. Thus, this conversion may not be accurate under very low shear conditions.

### RBC deformation in a multi-cell channel flow

While the Taylor deformation index has been used to describe the deformation of a single cell in simple shear flow, it cannot be used to represent the deformations of the multiple cells in a channel flow since the non-uniform strain rate in a channel flow produces eccentric deformations in the RBCs. Consequently, the determination of the major and minor axes for the eccentric-ellipse is subjective and prone to various interpretations. Hence, we propose the use of the cell perimeter to calculate the RBC membrane circumferential strain *ε* and the earlier introduced perimeter extension ratio *λ*_*C*_ to describe the overall deformation of the cells in the channel flow; *ε* is given by:

(21) $$\varepsilon =\lambda_{C}-1=\left(\sum \frac{l_{m}}{L_{0}}\right)-1$$

#### Model comparison, 2D-LD against 2D-neo-Hookean

**Figure 5:**
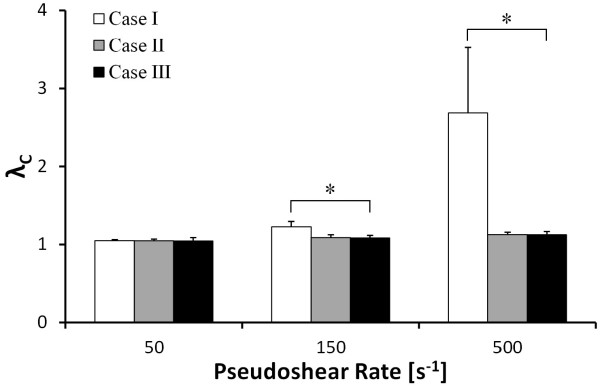
**Comparison of the perimeter extension ratio *λ***_***C***_** with varying degrees of the LD scaling coefficient application in Cases I, II and III.** *P < 0.001

**Figure 6:**
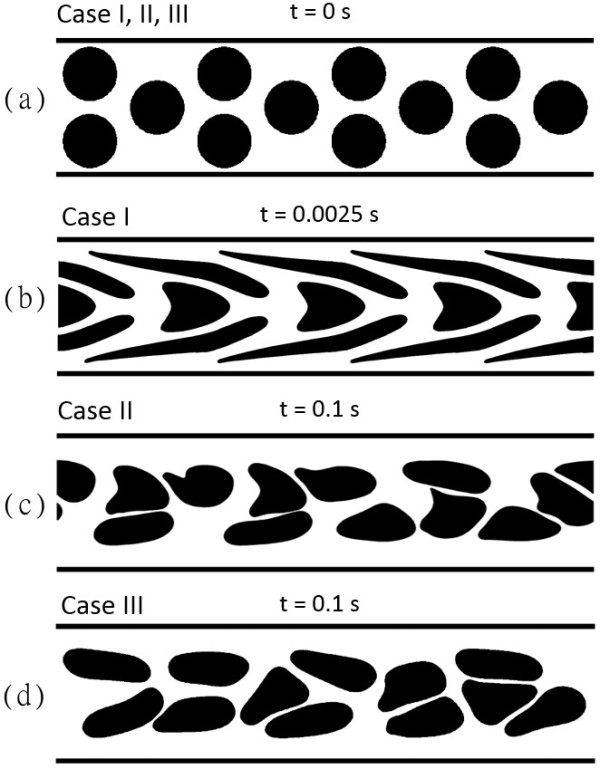
**Instantaneous snapshots of the RBC deformation profiles for the three cases under the highest pseudoshear rate of 500 s**^**-1**^**. (a)** Initial arrangement of RBCs for all three cases. **(b)** Simulated result from Case I. This case presents pronounced RBC stretching under the high shear condition whereby cells near the wall undergo “noodle-like” transformations. It should be noted that the simulation failed at t = 0.0025 s due to the non-physiological deformation of RBCs near the channel wall. **(c)** and **(d)** Simulated results from Case II and Case III. Case II exhibits higher incidences of “pinched” RBCs with sharp-edged profiles than those of Case III, thus demonstrating the effect of the bending treatment for large deformation applied in Case III but absent in Case II.

A comparison of the predicted RBC deformation between the three cases demonstrating the efficacy of the 2D-LD model is shown in Figure 5. The RBC deformation was quantified by taking the ensemble average of the 12 cells’ perimeter extension ratio *λ*_*C*_. At 50 s^-1^, there was no statistical difference among the three cases (*λ*_*C*_ = 1.049 ± 0.013 for Case I, 1.048 ± 0.002 for Case II, and 1.044 ± 0.043 for Case III). However, at 150 s^-1^, there was a ~13% difference (*P* < 0.001) in the average extension between Case I (1.225 ± 0.070) and Case III (1.083 ± 0.033), but no significant difference between Case II (1.088 ± 0.037) and Case III. Similarly at 500 s^-1^, there was a ~135% difference (*P* < 0.001) between Case I (2.688 ± 0.835) and Case III (1.125 ± 0.040), but no statistical difference between Case II (1.125 ± 0.031) and Case III. The pronounced difference in perimeter extension between Case I and the other two cases was expected since the LD model imposes a larger restrictive force on the membrane when it stretches beyond a stipulated limit. Thus, even in a very high shear condition of 500 s^-1^, the RBC perimeter does not extend by more than 12% of the original length for the 2D-LD model in Case III whereas Case I’s RBCs have stretched in length by more than two times of their original perimeter. Figure 6 shows the deformation profiles of the RBCs for Case I – III in the channel flow at a particular instant in time. As observed in Figure 6a, all three cases were initialized from the same symmetrical arrangement but the RBC flow developed differently with time (Figures 6b–d). Due to the over-extension of RBCs in the simulation, the RBC flow for Case I never reached a developed flow condition for the simulation conducted at the highest shear condition of 500 s^-1^ (Figure 6b). Subsequently, simulation failure occurred before the RBC flow structure could break its initial symmetric arrangement which typically occurs within 0.1 s of RBC flow as observed for Cases II and III in Figures 6c and d. Interestingly, the deformation profiles of cells observed in Case I for Figure 6b indicate that the extensive stretching of RBCs into “noodle-like” profiles occurs predominately for cells located in the high shear rate regions near the channel walls. From this evaluation of Case I’s result, it can be concluded that the 2D neo-Hookean model has a limitation in performing RBCs flows at high shear rates typical to microfluidic devices (> 1000 s^-1^). Through a comparative investigation of multi-cell simulations with (Cases II and III) and without (Case I) LD augmentation, we have established that LD augmentation is required for 2D RBC models to maintain physiological 2D RBC deformations, particularly for the cells travelling in close proximity to or impinged against the channel wall (see Figure 6b). A very recent study [13] on 2D multiple RBC flow simulations in a bifurcation also showed this limitation of the 2D RBC deformation simulation. They have illustrated that even at 100 s^-1^, non-physiologically over-stretched RBC shapes (“noodles”) were obtained in the model simulation due to wall impingement and multi-directional strain near flow bifurcation corners, similar “noodling” of RBCs *in vivo* has not been reported.

While the presentation of deformation data in Figure 5 indicates that the overall perimeter remains statistically the same in Case II and Case III, the shapes of the RBCs were considerably different in these two LD-applied cases as evidenced by the images in Figures 6c and d. This may be due to the lack of strain-hardening on the remaining three constitutive models for the RBCs in Case II (as summarized in Table 1). The implication of this omission in Case II will be discussed in the following subsection.

#### Significance of bending resistance and contact forces for large deformations

**Figure 7:**
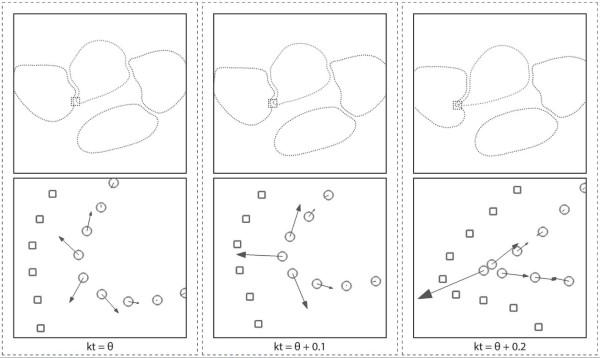
**Instantaneous profiles of the RBC membrane buckling under channel flow conditions.** The progression of the membrane pinching is shown in sequential frames as indicated by the dimensionless time *kt. θ* denotes the time at which the onset of pinching was observed. The bottom panel of images provide a zoomed-in view of the pinched region where an increasing force in the adverse direction contributes to the growing instability that finally leads to membrane buckling and twisting.

**Figure 8:**
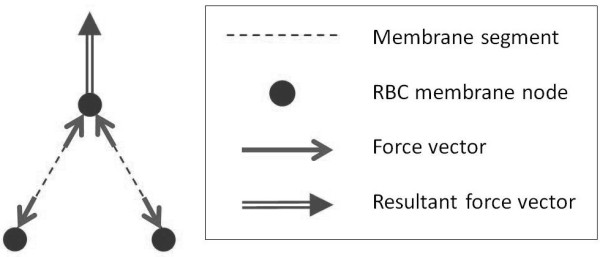
**Schematic diagram of the internal forces in the membrane segments that gives rise to a resultant force at each membrane node.** Under a combination of high compression and sharp curvatures between two membrane segments, the resulting force on the node shared between the segments can have a very large normal component, leading to an unrestricted “pinching” of the RBC membrane.

As mentioned earlier, our RBC model has a bending resistance modality that maintains the curvature of the RBC membrane. In Case II, the LD scaling coefficient was applied only to the membrane shearing resistance, while the cell-to-cell interaction forces and the bending resistance were left un-scaled in the simulation model. This means that while the constitutive bending behavior of the RBC and contact mechanics between cells are included in Case II’s model, their influence on the RBC membrane deformation diminishes with the increasing strain since only the shear component is augmented for strain-hardening. Eventually, at large deformation conditions, shear forces dominate the entire deformation behavior of the membrane in Case II. From the comparison between the mechanical constitutions in Case II and Case III, we can observe in Figures 6c and d that simply applying strain-hardening for the membrane shear stiffness alone without scaling the other constitutive moduli might generate an imbalance in the internal energies of the membrane that leads to physical instability of the membrane deformation. Accordingly, the results from Case II indicate a regular occurrence of the membrane buckling phenomenon. Figure 7 shows the instantaneous snapshots of the RBC membrane in the various stages of buckling. The increasing force vectors on the RBC membrane acting in an adverse direction leads to a compounding instability. This manifests as a twisting and apparent “pinching” of the membrane, leading to simulation failure. The cause of this instability is the high compressive forces that build up in the progressively shortened membrane segments in the pinched region of the membrane. This is portrayed in Figure 8 where the resultant force of two compressed segments calculated from the membrane shear model is exerted in the direction opposite to the spontaneous curvature. Without scaling the bending force to counter this large shear force, the membrane is allowed to buckle into non-physiological shapes with pinched areas of sharp curvature. Additionally, as the RBC-to-RBC interaction forces are not scaled, RBCs can impinge into one another due to insufficient repulsion, thus resulting in pairing membranes penetrating and overlapping each other. Conversely, when the three other constitutive models were scaled with the LD scaling coefficient as done so for the simulations performed in Case III, these two scenarios for membrane instability were successfully avoided. The comparison of RBC shapes and the differences in membrane curvature stability between the models implemented in Case II and III therefore highlight a major advantage of the present 2D-LD model. Unlike other non-linear models for large membrane deformation that only augment the membrane shear response, the large deformation coefficient *α* used in the 2D-LD model is a simple multiplicative operator that can be used to apply the same order of strain hardening to all elastic moduli involved in the RBC membrane’s constitutive response to deformation.

#### Cell-free layer width and relative apparent viscosity

The cell-free layer (CFL) and its role in influencing the apparent viscosity of blood is an important characteristic in quantifying microvessel and microchannel flows. Due to the shear-induced migration of RBCs towards the center of the vessel, the formation of a CFL along the vessel wall significantly lowers the apparent viscosity of blood in microvessel flows when compared against the uniform bulk viscosity of blood [1]. Accordingly, we validated our channel flow simulations by comparing the CFL width and the apparent viscosity predictions of our 2D-LD model in Case III (for the pseudoshear rate of 50 s^-1^) against the earlier work of Zhang and coworkers [29,30]. Our predicted CFL width was ~26% of the total channel width which is in good agreement with the value (27% – 32% at 58 s^-1^) reported in their study [29] where the simulation condition was similar to ours.

The apparent viscosity *μ*_*app*_ of blood in our channel flow simulations was calculated using the Poiseuille formula:

(22) $$\mu_{\mathrm{app}}=\frac{\mathrm{\Delta P}H^{3}}{12QL_{\mathrm{channel}}}$$

where Δ*P* is the pressure difference applied across the channel length *L*_*channel*_, *H* is the channel width and *Q* is the resulting flow rate. For comparison against the literature, the apparent viscosity was normalized by the plasma viscosity *μ*_*plasma*_ to provide the relative apparent viscosity *μ*_*rel*_:

(23) $$\mu_{\mathrm{rel}}=\frac{\mu_{\mathrm{app}}}{\mu_{\mathrm{plasma}}}\;$$

Our result (1.10) falls within the range of the simulated results by Zhang and colleagues: 1.05 in a 20-μm channel [30] and 1.29 in a 12-μm channel [29]. Although the comparisons of the CFL width and the relative apparent viscosity indicate reasonable agreement between our results and theirs, it should be noted that circular RBC profiles were considered for our 2D flow model while they have represented the 2D flow of RBCs using biconcave RBC profiles. Subsequently, even though we have a higher 2D hematocrit of 38% in comparison to their 30.5% [29], our actual number of RBCs in the simulation is much fewer (12 circular RBCs vs. 27 biconcave RBCs). As a result of this, it may be limited to directly compare our relative apparent viscosity and cell-free layer width with the values reported by them.

**Figure 9:**
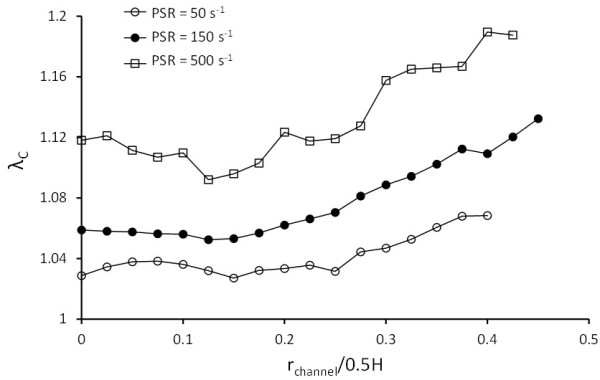
**Relation between cell perimeter extension ratio *λ***_***C***_** and its transverse location r**_**channel **_**in the channel.** RBCs further away from the channel centerline undergo larger deformations due to the higher shear rates near the channel walls.

It is of note that the CFL width and relative apparent viscosity are dependent on rheological factors such as the pseudoshear rate, hematocrit and channel width. In the present study, we have considered only a single channel configuration with a width of 20 μm and a hematocrit of 38% under various pseudoshear rates. Hence, our analysis of the RBC dynamics in terms of the CFL width, apparent viscosity and RBC deformation may be limited to the present channel configuration. In accordance with the Fahraeus-Lindqvist effect, the CFL width as a fraction of the channel width (fractional CFL width) is expected to increase with a reduction in channel width as reported in the earlier work by Kim and et al. [36]. With regards to the RBC deformation, when the channel width is increased, the corresponding decrease in the fractional CFL width would result in an increase in the RBC perimeter extension ratio *λ*_*C*_. This is in accordance with the result shown in Figure 9 where the RBC deformation increases when the distance between the RBC and the channel wall is reduced.

#### RBC stress and strain

A number of previous studies [1,28,37-39] have used pseudoshear rate in replacement of the actual shear rate to quantify the shear condition on RBCs in microvessel flows. This assumption does not hold since RBCs travelling near the channel wall experience higher shear stress (strain) than RBCs located in the center of the channel. Consequently, evaluation of *G* using the pseudoshear rate for *k* in Eq. (19) could be a poor representation of the actual shear condition applied on the RBCs in a channel flow. Therefore, in this study, we introduce a new dimensionless shear rate parameter G* to provide a better approximation of the local shear condition in the channel flow as follows:

(24) $$G^{*}\left({\vec{r}}_{\text{min}}\right)=\frac{\tau_{\text{max}}\left({\vec{r}}_{\text{min}}\right)L_{C}}{E_{s}}$$

where *τ*_max_ is the time-averaged maximum shear stress on the RBC membrane and it is a function of the the minimum RBC membrane to channel wall distance ${\vec{r}}_{\text{min}}$.*τ*_max_ was obtained by recording the maximum shear stress exerted on the RBC membrane for each successive time-step and thereafter performing a time-averaging calculation on the maximum stress over the period of analysis. In the present study, we have analyzed the RBC mechanics over 10 material transit cycles. The material transit time represents the average time it takes for a cell entering the simulation domain to exit at the channel outlet and this was estimated using the bulk flow velocity and the channel length. Since both *τ*_max_ and the cell perimeter *L*_*C*_ are dependent on the RBC trajectory in the channel, G* can provide a closer approximation of the spatially and temporally varying shear condition than the traditional *G* for a channel flow study.

**Figure 10:**
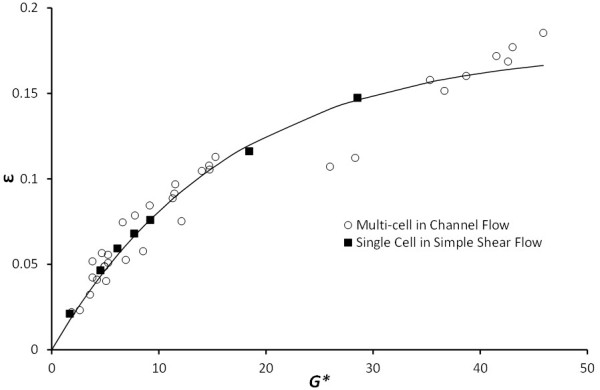
Relation between cell circumferential strain*ε*and the local dimensionless shear rateG*. A nonlinear regression fit of results for the single cell in simple shear flow and multi-cells in channel flow indicates the validity of the *ε* and G* characterizations for general shearing conditions on the 2D RBC profile $\left(\varepsilon =-0.1771e^{-0.0611G*}+0.1771;R_{\mathrm{Single}}^{2}=0.996\;\mathrm{and}\;R_{\mathrm{Multi}}^{2}=0.939\right)$.

With this G*, the deformation state of RBCs in a general flow condition can be characterized and compared by using the relation between G* and the membrane circumferential strain *ε*. To prove the validity of the G* and membrane strain relation, both parameters were calculated for the two different simulation sets performed in this study. In Figure 10, the relation between the two parameters is compared for the single-cell in simple shear and the multi-cells in channel flow conditions. An exponential curve (*ε* = – 0.1771*e*^-0.0611G*^ + 0.1771) was fitted by non-linear regression to both the single cell deformation results and multi-cell deformation results to obtain the empirical expression for circumferential strain against varying G*. The regression curve produced high *R*^*2*^ values of 0.996 and 0.939 for the simple shear and channel flow data sets respectively, indicating that the two different shear conditions can be described by the same relation between G* and *ε*. This implies that by using G* and *ε*, we can relate the single-cell simple shear flow results to the multi-cell channel flow results.

On the other hand, using the original dimensionless shear rate *G* to relate between the two sets of flow conditions demonstrates the limitation of the *G* parameter as a universal tool for relating the applied shear to the RBC elastic resistance. RBCs in a channel flow under the pseudoshear rate of 150 s^-1^ (*G* = 0.125) exhibited a wide range of deformation *ε* (0.05 – 0.11) that is dependent on the cell location as seen in Figure 9 where the RBC perimeter extension ratio *λ*_*C*_ was plotted against RBC center of mass location. Corresponding to this range of *ε*, the RBC in a simple shear flow exhibited similar values of 0.05 and 0.11 when the applied *G* was 0.2 and 1.0, respectively. Hence, it can be concluded that while the stress-strain relation between *G* and *ε* is valid for the simple shear flow, it is weak for the case of channel flows since a single value of *G* presents multiple strain possibilities depending on the cell location in the channel (Figure 9).

The case for using G* presented in Figure 10 to signify RBC stress-strain behavior becomes particularly favorable when we wish to provide a more accurate estimation of shear stress acting on the RBC at high shear rates. Using Eq. (24), we can obtain *τ*_max_ acting on the RBC in experiments by obtaining circumferential strain *ε* and RBC perimeter *L*_c_ through imaging and measurement techniques and G* by reading off its corresponding value against *ε* in Figure 10. Therefore, Figure 10 and Eq. (24) can be used to predict the maximum shear stress on an RBC for a given profile in the channel flow for experimental studies where shear stress cannot be obtained through direct measurement of the shear stress quantity. For our particular study, the maximum pseudoshear rate considered was 500 s^-1^ for a channel of 20-μm width. The corresponding time-averaged maximum shear stress on the RBCs in the flow was approximately 10 Pa (G* = 47). This is well below the reported shear stress (300 Pa for 120 s of shear) for RBC lysis [40] or even sub-hemolytic damage to the RBC membrane [41]. Hence, even though strain hardening is expected of the RBC at shear rates > 500 s^-1^, this is by no means suggestive of mechanical damage to the RBC.

### Potential limitations of the 2D-LD model

One possible limitation of our 2D-LD model can arise from the chosen 2D RBC profile for the channel flow simulation. Firstly, a similar simulation on the 2D biconcave RBC profile may give a different set of results due to its higher bending and flexing capability than the circular RBC. While the LD scaling model will not be different in form for biconcave cells, a calibration of the model terms and coefficients would be required. Furthermore, we have assumed that the cross-sectional area remains constant in the 2D plane of investigation. This is necessary for the 2D model to maintain the channel hematocrit and to enforce the 2D conservation of mass. This model is therefore limited to flow situations where 2D RBCs remain in the plane of observation. However, such flow conditions can easily be found in most microfluidic flows where the Reynolds number is very low.

Additionally, our present 2D-LD model lacks the inclusion of the membrane viscosity and its effect on the dynamic deformation behavior of RBCs. It is likely that the membrane viscosity will affect the dynamic behavior of RBCs that are subjected to ever-evolving shear rates due to the variation in their transverse location as they travel along their respective trajectories within the channel. Membrane viscosity is likely to delay the deformation response of RBCs to fluctuations in the shearing condition as a result of the changes in RBC position and orientation. However, our present model does include the effect of cytoplasmic viscosity (6.0 cP) and the plasma viscosity (1.2 cP), and our earlier work [27] has shown that the inclusion of the difference in viscosity between the two fluids can similarly delay the RBC membrane deformation response.

## Conclusion

In the present study, we have presented a 2D large deformation (2D-LD) model to augment the elastic moduli of the RBC membrane in the high shear rate flow regimes. The efficacy of the model and the predictive accuracy of the resulting 2D deformation states were tested on a single circular RBC profile under a simple shear condition and the results were found to be in good agreement with the 3D data. Furthermore, this study highlights the importance of sufficiently scaling the various membrane mechanics models to prevent numerical instabilities in the simulation. In our analysis of the stress-strain relation for the membrane, we have also proposed a new dimensionless shear rate term G* to generalize the shear condition on a RBC so as to predict the extent of deformation regardless of flow conditions.

Therefore, our 2D-LD model can be applied to blood flows in practical microfluidic studies involving channel bifurcations and cell mechanical partitioning [37] where high and multi-direction strain can be applied on the RBCs at flow dividing locations. These studies would need robust mechanical models to predict the RBC deformation without incurring the high computational cost that is generally required for 3D simulations. Thus, by utilizing our LD model, it would be possible to simulate a blood flow in a microfluidic system, and such a model would enable us to optimize a microfluidic channel structure for biomedical applications at relatively low computing cost.

## Competing interests

The authors declare that they have no competing interests.

## Authors’ contributions

SSY was the primary investigator who conceived the idea, formulated the model, wrote the code, performed the analysis and wrote the paper. YCN and JT implemented and ran the simulations, revised the key modelling coefficients, and assisted in the initial drafts of the paper. HLL reviewed the process of developing the model and the discussion of our findings. SK was the lead investigator who supervised the work content and modeling strategy. All authors read and approved the final manuscript.
